# Milrinone versus dobutamine in acute myocardial infarction-related cardiogenic shock; a propensity score matched analysis

**DOI:** 10.1007/s00392-025-02742-0

**Published:** 2025-09-03

**Authors:** Sanne ten Berg, Margriet Bogerd, Elma J. Peters, Marijke J. C. Timmermans, Wim K. Lagrand, Luuk C. Otterspoor, Alexander P. J. Vlaar, Annemarie E. Engström, José P. S. Henriques, the PCI Registration Committee of the Netherlands Heart Registration, J. M. Cheng, M. Meuwissen, M. Grundeken, R. Al Hashimi, K. Teeuwen, M. Magro, R. Diletti, B. J. Sorgdrager, C. E. Schotborgh, R. J. R. Snijder, J. Polad, R. Scherptong, E. Bakker, A. J. W. van’‘t Hof, F. Spano, J. Brouwer, K. G. van Houwelingen, Robyn McClelland, J. van Ramshorst, G. Amoroso, C. Camaro, P. W. Danse, K. Sjauw, E. K. Arkenbout, W. T. Ruifrok, A. O. Kraaijeveld, E. Lipsic, L. Hoebers, R. Erdem

**Affiliations:** 1 Department of Cardiology, Amsterdam, UMC, Meibergdreef 9, 1105 AZ Amsterdam, The Netherlands; 2 Netherlands Heart Registration, Moreelsepark 1, 3511 EP Utrecht, The Netherlands; 3 Department of Intensive Care Medicine, Amsterdam UMC, Amsterdam, The Netherlands; 4 Heart Centre, Catharina Hospital Eindhoven, Eindhoven, The Netherlands

**Keywords:** Myocardial infarction, Shock, Cardiogenic, Milrinone, Dobutamine

## Abstract

**Background:**

Vasopressors and inotropes remain the cornerstone in treatment of acute myocardial infarction-related cardiogenic shock (AMI-CS). Milrinone and dobutamine are both commonly used, yet the optimal inotrope remains unclear. We aimed to identify factors associated with milrinone and dobutamine treatment and assess their effects on 30-day mortality in a large real-world cohort of AMI-CS patients. The Netherlands Heart Registration prospectively records data for percutaneous coronary intervention patients. Between 2017 and 2021, additional retrospective data on CS patients were collected by fourteen Dutch hospitals. Patients who were treated with either milrinone or dobutamine were selected; those treated with both were excluded. Missing data were imputed (30 ×) using multiple imputation, and propensity matched score analysis (PSM) was performed to evaluate the association between milrinone or dobutamine treatment and 30-day mortality.

**Results:**

In total, 739 patients were included (milrinone *n* = 247, dobutamine *n* = 492). Prior to matching, milrinone-treated patients exhibited more severely ill baseline and treatment characteristics, and higher 30-day mortality (50.6% vs. 41.5%, *p* = 0.018). After PSM, 198 patients remained in each group for analysis. Baseline characteristics were well balanced and 30-day mortality rates were similar (46.5% vs. 41.9%, *p* = 0.362).

**Conclusion:**

In this real-world propensity-matched cohort of AMI-CS patients, no significant difference in 30-day mortality was observed between patients treated with milrinone and dobutamine. Importantly, milrinone patients were more severely ill at baseline, indicating that the choice of inotrope may be influenced by illness severity. This comprehensive study suggests that the selection of inotrope may continue to be guided by individual patient characteristics.

**Graphical Abstract:**

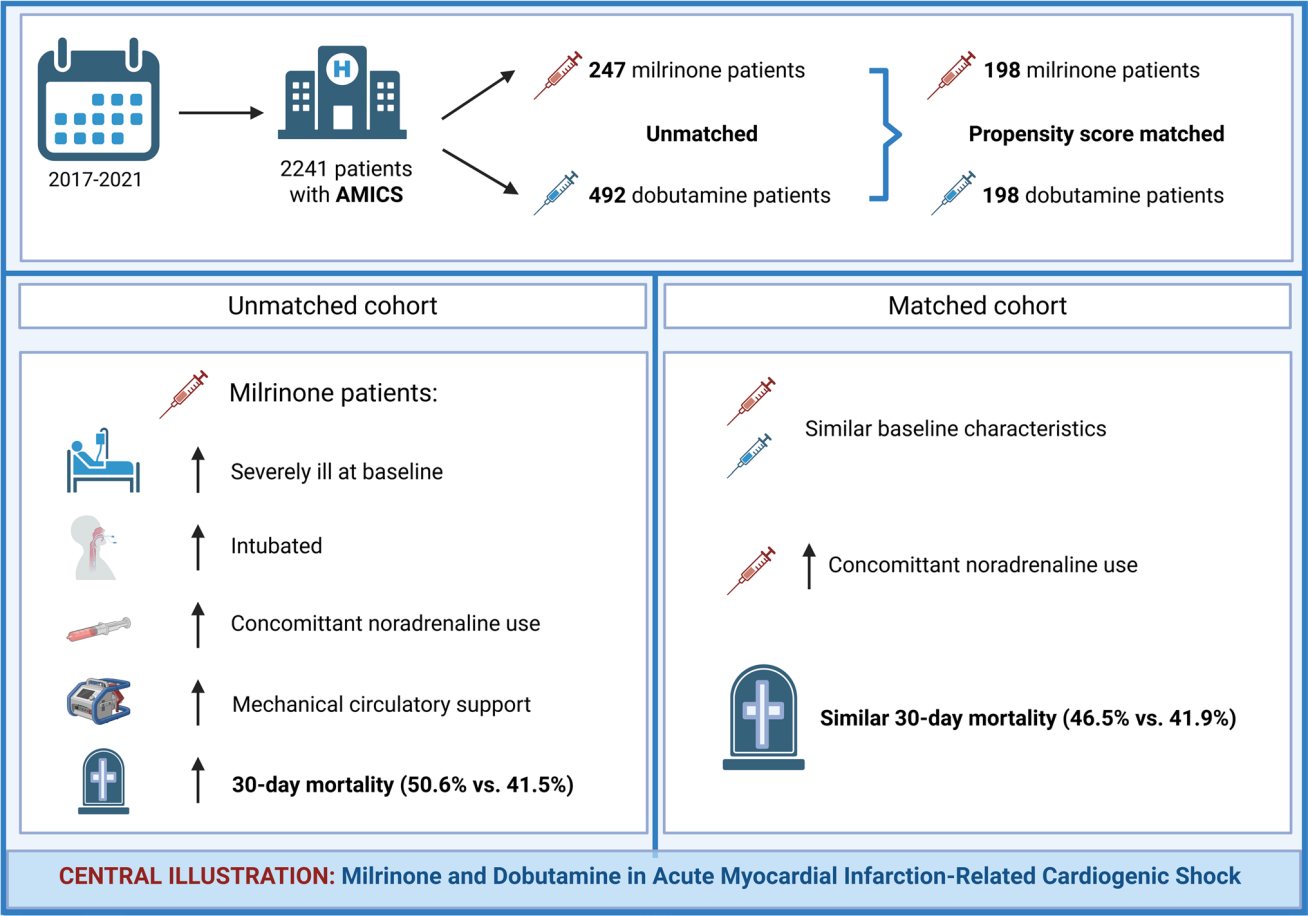

**Supplementary Information:**

The online version contains supplementary material available at 10.1007/s00392-025-02742-0.

## Introduction

Cardiogenic shock (CS) is characterized by a clinical presentation of end-organ hypoperfusion, requiring intervention with vasoactive medication or mechanical circulatory support (MCS) [1, 2]. Although CS complicates only 3–13% of acute myocardial infarction cases (AMI), it remains the leading cause of death [2–6]. Despite multiple attempts to improve treatment strategies, mortality rates in acute myocardial cardiogenic shock (AMI-CS) have remained persistently around the 40–50% for years [4, 5, 7–9]. Two decades ago, the SHOCK trial demonstrated lower 6-month mortality after immediate revascularization in AMI-CS patients [10]. Recently, the DANGER shock trial reported a significant survival benefit with the microaxial flow pump, in a highly selected cohort of non-comatose AMI-CS patients with ST-elevation myocardial infarction (STEMI) [11].

Nevertheless, vasopressors and inotropes remain the cornerstone of treatment for CS. Both the European and the American guidelines recommend noradrenaline as the first-line vasopressor, based on two randomized controlled trials (RCTs). comparing noradrenaline to both dopamine and epinephrine, but no recommendation is made on the inotrope of choice [1, 12–14].

Dobutamine and milrinone are both frequently used as inotropes in the treatment of CS. Dobutamine is a synthesized catecholamine, stimulating mainly β1 receptors, exhibiting mild vasodilatory effects from β2 stimulation and α1 antagonism at low doses. Milrinone, on the other hand, a phosphodiesterase inhibitor, has inotropic and lusitropic effects, causing more pronounced hypotension due to increased vasodilation. Unlike dobutamine, milrinone does not stimulate β-receptors, lowering the risk for tachycardia and the accompanying increase of myocardial oxygen consumption [15, 16]. This offers a theoretical advantage in preventing myocardial ischemia and arrhythmias, particularly of importance in AMI-CS patients.

The DOREMI trial (*n* = 192) is the only RCT comparing the effects of dobutamine and milrinone in a CS population and reported no significant difference in primary or secondary clinical outcomes [17]. However, since the DOREMI trial included all-comers with CS, with only a third of the participants (*n* = 65) being AMI-CS patients, the effects of these two inotropes on outcomes in AMI-CS remain unclear. Therefore, we aimed to identify patient characteristics associated with treatment with milrinone and dobutamine and to assess their effects on 30-day mortality in a large real-world cohort of AMI-CS patients.

## Methods

### Study design

This observational, retrospective multicenter study utilized prospectively collected data from the Netherlands Heart Registration (NHR). The NHR is a nationwide, physician-driven and patient-focused quality registry. Within the NHR, baseline, procedural, and outcome data are collected [18, 19]. This study was approved by the institutional review board Medical Research Ethics Committees United (W19.270), and a waiver for informed consent was granted.

### Data collection and study population

All patients in the Netherlands undergoing percutaneous coronary intervention (PCI) are prospectively registered in the NHR. From January 2017 to September 2021, the registry was expanded with an additional variable set for patients who were in CS. These additional data were collected by 14 hospitals. Further details on the process of data collection have been described previously [20]. Data on vasopressor and inotrope administration, initiated either before PCI or within 24 hours post PCI, were collected, including noradrenaline, adrenaline, dopamine, dobutamine, and milrinone. Patients with missing data for all vasopressors and inotropes were excluded. Patients who were treated with either dobutamine or milrinone were selected, and patients who were treated with both were excluded from the main analyses. For practical reasons ‘milrinone’ refers to treatment with either milrinone or enoximone.

### Endpoints

The primary endpoint comprised all-cause 30-day mortality. Mortality status was retrieved from the governmental Personal Records Database. The clinical course was compared between the milrinone and dobutamine groups based on the following process variables: concomitant use of other vasopressors or inotropes, post-PCI thrombolysis in myocardial infarction (TIMI)-flow (0–2 or 3), use of MCS, lactate level post PCI (mmol/L), troponin (ng/L), and CKMB levels (mcg/L) (highest level measured within 1–3 days after admission), left and right ventricular ejection fraction (LVEF and RVEF, in %), and hospital length of stay (LOS) in days.

### Statistical analysis

#### General

Continuous baseline data were presented as either means and standard deviations (*SD*s) in case of normally distributed variables or medians and interquartile ranges (*IQR*s) in case of non-normally distributed variables. Normally distributed data were compared with a two-sample *t*-test, while non-normally variables were analyzed with the Mann–Whitney *U* test. Categorical data were presented as counts and percentages and were compared using the chi-squared/Fisher’s exact test. Propensity-score matching (PSM) was performed to assess the association between treatment group and the outcome in adjustment for confounding. Mortality at 30 days was analyzed using the Kaplan–Meier method for both the matched and unmatched cohorts, with reporting of log-rank statistics, hazard ratios (*HR*s) and corresponding confidence intervals (*CI*s). A *p*-value < 0.05 was considered statistically significant for all analyses. All statistical analyses were performed using R statistical software (v4.3.2 R Core Team 2023).

#### Imputation

Data were imputed using multiple imputation to account for missing data. Irrelevant variables, variables containing more than 30% missing data, constant and collinear variables were removed prior to imputation to minimize bias in the imputation process. The imputation was performed using the predictive mean matching method with the MICE package v3.16.0 in R [21]. A total of 30 imputed datasets were generated, ensuring robust estimates across the imputations.

#### Unmatched illness severity

To compare unmatched illness severity of both groups, the Acute Coronary Syndrome in Cardiogenic Shock (ACCS) risk score was applied [22]. Using the regression formula from Model 2, predicted 30-day mortality was calculated for each patient across 30 imputed datasets. The average scores were then merged with the original dataset, and group means were compared using a *t*-test.

#### Propensity score matching

To address potential confounding, we utilized average PSM to compare 30-day mortality and treatment effects of milrinone versus dobutamine. Covariates for the PSM model were selected from variables known at admission, along with angiographic and treatment characteristics assessed at the time of PCI. Selected covariates can be found in Supplemental Table S1.

Propensity scores were calculated per patient across the 30 imputed sets using the R-package MatchThem v1.1.0, with nearest neighbor matching applied within a caliper of 0.10. Quadratic terms were added for all continuous variables to improve balance. These propensity scores were then averaged, and matching was subsequently performed in the original dataset using the MatchIt function to achieve a 1:1 ratio of milrinone to dobutamine patients. Balance was assessed by the standard mean difference (*SMD*), with an *SMD* < 0.1 indicating adequate balance.

#### Subgroup analyses

Subgroup analyses were performed in the matched cohort by logistic regression models to evaluate differences in 30-day mortality across subgroups. These subgroups were based on sex (male/female), age (above or below 65 years), body mass index (*BMI)* (above or below 30 kg/m²), diabetes (yes/no), baseline renal function (eGFR above or below 60 ml/m^2^), presentation with an out-of-hospital cardiac arrest (OHCA) (yes/no), etiology of acute coronary syndrome (STEMI or nSTEMI), lactate at admission (above or below 4.5 mmol/l), intubation before admission to the coronary care unit (CCU) or intensive care unit (ICU) (yes/no), presence of multivessel disease (MVD) (yes/no), PCI of the right coronary artery (RCA) (yes/no) and MCS (yes/no). The *p*-values represent an interaction *p*-value, indicating whether the association between treatment with milrinone or dobutamine and 30-day mortality is different between the subgroups.

#### Additional analyses

To provide broader clinical context, an unmatched baseline table including patients treated with both milrinone and dobutamine, and without any inotrope, was added to the supplementary material. In the ‘no inotrope’ group, patients treated with dobutamine, milrinone, adrenaline, and dopamine were excluded, leaving patients treated solely with noradrenaline or without any inotrope. To further explore outcomes, an unmatched Kaplan–Meier analysis was performed. Additionally, a Kaplan–Meier sensitivity analysis of the matched cohort was conducted for patients without an OHCA or in-hospital cardiac arrest (IHCA). As an exploratory analysis, 30-day mortality was compared between patients treated with extracorporeal membrane oxygenation (ECMO) versus Impella, stratified by milrinone and dobutamine group.

## Results

### Study cohort

**Fig. 1:**
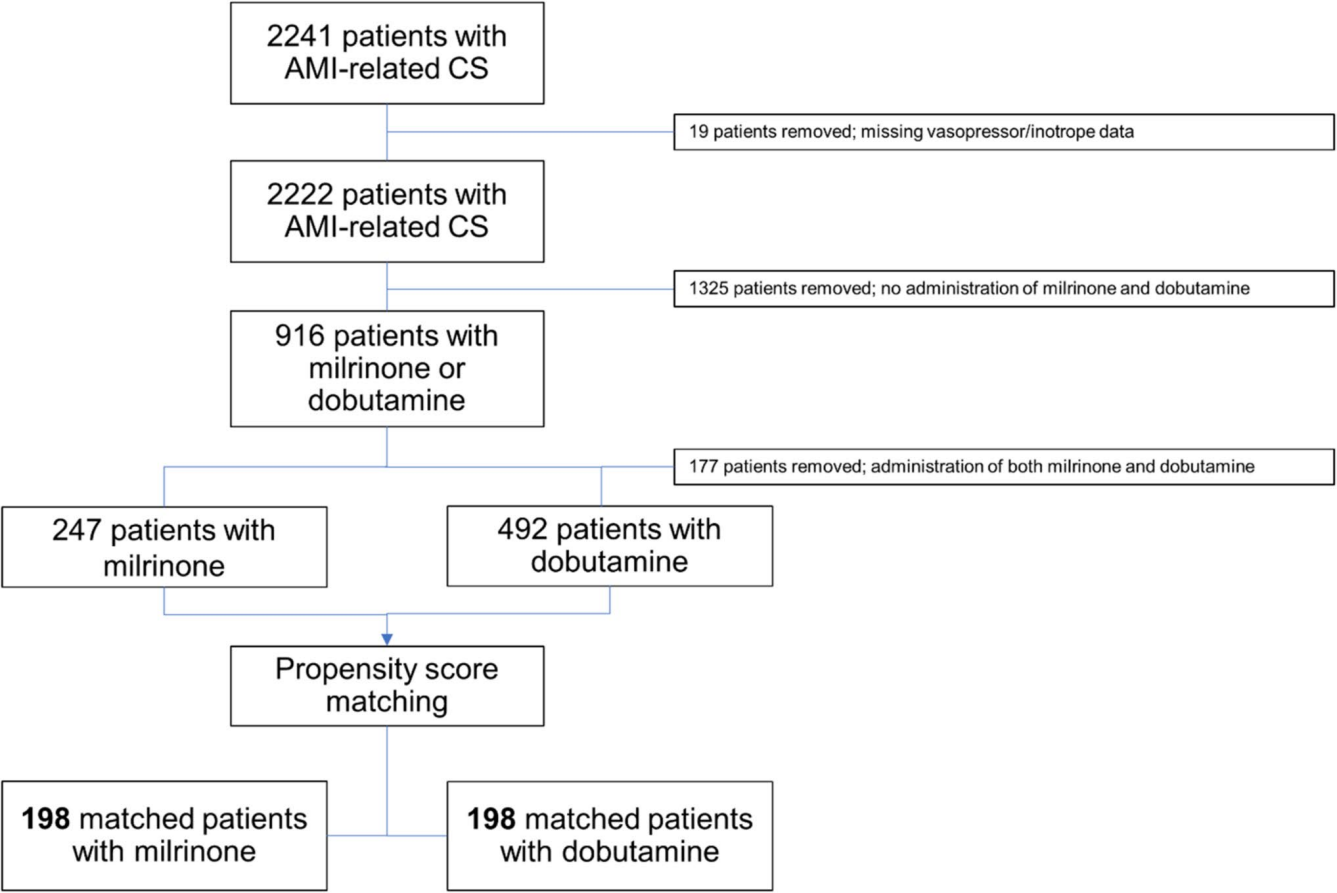
Study flow chart

The total study cohort consisted of 2222 AMI-CS patients with available vasopressor and inotrope data, of whom 916 (41.2%) received milrinone, dobutamine, or both, as shown in Fig. 1. A total of 177 patients were treated with both milrinone and dobutamine and were excluded from further analyses. As a result, the cohort for the current study consisted of 739 patients treated with either milrinone (*n* = 247) or dobutamine (*n* = 492). After PSM, a total of 396 patients were available for analysis; 198 patients in each group.

In the unmatched cohort, the mean age was 66.7 (± 12.1) years and 69.8% (*n* = 516) of the patients were male (Table 1). At baseline, patients treated with milrinone had a higher *BMI* and were more likely to have experienced a prior coronary event than those treated with dobutamine. Upon admission, milrinone patients presented with a higher heart rate, presented with NSTEMI more often, and had a symptom duration exceeding 3 hours more frequently. Also, milrinone patients were more often intubated prior to CCU or ICU admission. Moreover, these patients had lower levels of hemoglobin, but higher creatinine levels upon admission. At the catheterization laboratory, milrinone patients were more likely to undergo PCI of the ramus circumflex. Using the ACCS-risk score to assess baseline illness severity, the predicted 30-day mortality was 0.515 in the milrinone group and 0.475 in the dobutamine group, with a *p*-value of 0.025.

**Table 1:** Baseline table unmatched and matched cohort

Characteristics	Unmatched cohort				Matched cohort		
	Milrinone, *N* = 247^a^	Dobutamine, *N* = 492^a^	*p***-**value	Missing %	Milrinone, *N* = 198^a^	Dobutamine, *N* = 198^a^	*SMD*^b^
Baseline characteristics							
Age	66.0 (12.0)	67.1 (12.1)	0.235	0	65.8 (12.5)	65.9 (12.5)	0.01
Gender, male	177 (71.7%)	339 (68.9%)	0.441	0	136 (68.7%)	139 (70.2%)	0.03
* BMI*	27.7 (5.0)	26.6 (4.5)	0.013	14	27.2 (4.2)	27.3 (4.7)	0.03
Diabetes	59 (24.6%)	108 (23.0%)	0.633	3.9	45 (23.6%)	43 (23.1%)	0.01
Prior event	85 (37.1%)	126 (28.8%)	0.028	9.7	61 (33.5%)	58 (32.2%)	0.03
Admission characteristics							
MAP (mmHg)	77.7 (22.6)	77.8 (24.1)	0.947	13	78.1 (23.0)	79.0 (24.5)	0.04
Heart rate (bpm)	95.9 (27.5)	82.5 (27.6)	< 0.001	14	92.4 (27.2)	92.5 (27.4)	0.01
STEMI (vs. NSTEMI)	178 (72.1%)	425 (86.4%)	< 0.001	0	163 (82.3%)	160 (80.8%)	0.04
Symptoms > 3 h	120 (56.1%)	169 (40.0%)	< 0.001	14	87 (49.7%)	85 (50.6%)	0.02
OHCA	116 (47.2%)	201 (41.0%)	0.113	0.4	93 (47.2%)	92 (46.7%)	0.01
Witnessed arrest	80 (33.1%)	167 (34.6%)	0.670	2.0	72 (37.3%)	71 (36.8%)	0.01
Resuscitation duration ≥ 30 min	32 (14.0%)	55 (11.7%)	0.654	5.1	28 (15.1%)	26 (13.8%)	0.04
IHCA	16 (6.5%)	28 (5.7%)	0.685	0.4	11 (5.6%)	14 (7.1%)	0.06
Intubated before CCU/ICU admission	168 (68.6%)	274 (56.4%)	0.001	1.1	131 (66.8%)	129 (66.2%)	0.01
Laboratory values at admission							
Lactate (mmol/l)	5.6 (2.6, 9.5)	6.0 (3.0, 9.6)	0.243	22	5.6 (2.7, 9.4)	5.8 (2.8, 9.8)	0.05
Hemoglobin (mmol/l)	8.0 (1.6)	8.3 (1.4)	0.028	3.8	8.2 (1.4)	8.2 (1.4)	0.05
Glucose (mmol/l)	12.8 (8.6, 18.7)	13.4 (9.4, 17.8)	0.203	9.3	13.2 (9.1, 19.0)	13.3 (10.2, 17.3)	0.01
Creatinine (μmol/l)	111.0 (88.0, 139.0)	103.0 (82.0, 123.0)	0.002	8.7	106.0 (85.0, 134.0)	106.0 (88.0, 130.0)	0.07
Angiography							
Left main	47 (19.0%)	76 (15.4%)	0.218	0	37 (18.7%)	29 (14.6%)	0.11
LAD	110 (44.5%)	214 (43.5%)	0.788	0	87 (43.9%)	91 (46.0%)	0.04
RCX	71 (28.7%)	92 (18.7%)	0.002	0	51 (25.8%)	54 (27.3%)	0.03
RCA	74 (30.0%)	142 (28.9%)	0.757	0	58 (29.3%)	57 (28.8%)	0.01
Pre-TIMI flow 2–3	66 (31.1%)	96 (23.7%)	0.046	17	49 (28.8%)	40 (25.2%)	0.08
Multivessel disease	164 (66.4%)	299 (61.1%)	0.164	0.4	131 (66.2%)	127 (64.5%)	0.04

*BMI* body mass index, *CCU* critical care unit, *ICU* intensive care unit, *IHCA* in-hospital cardiac arrest, *LAD* left anterior descending artery, *MAP* mean arterial pressure, *NSTEMI* non-ST-elevation myocardial infarction, *OHCA* out-of-hospital cardiac arrest, *RCA* right coronary artery, *RCX* ramus circumflex, *STEMI* ST-elevation myocardial infarction, *TIMI-flow* thrombolysis in myocardial infarction

^a^Mean (SD); n (%); median (Q1; Q3)

^b^Standardized mean difference

After PSM, all baseline, admission, and treatment characteristics assessed at the time of PCI were well balanced, except for a slight imbalance in the prevalence of the left main as the culprit (SMD 0.11).

### Outcomes in the unmatched cohort

**Fig. 2:**
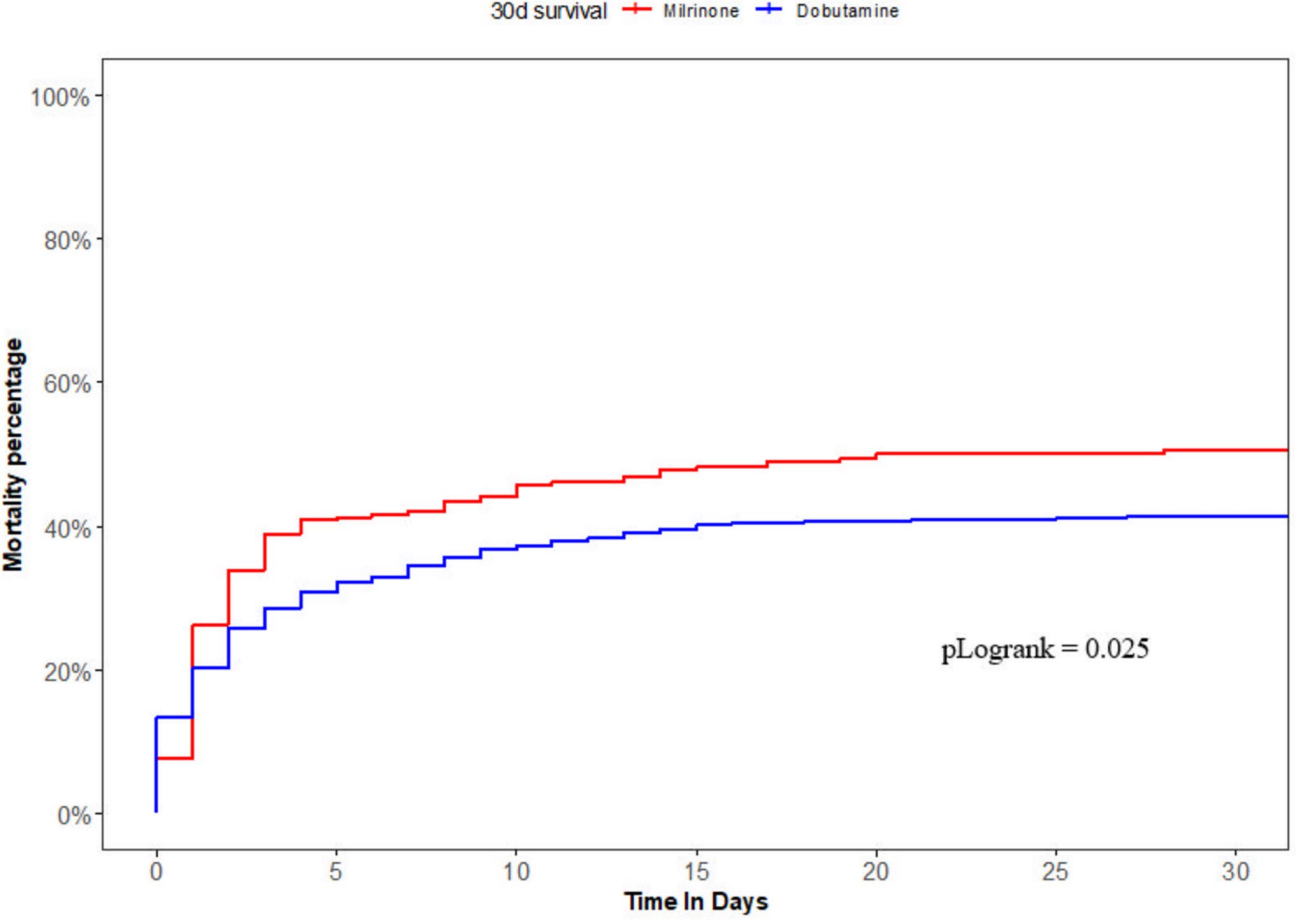
Kaplan–Meier curves of the unmatched cohort

In the unmatched cohort, 30-day mortality rates were higher in patients treated with milrinone (50.6% vs. 41.5%, *p* = 0.018), with a corresponding unadjusted *HR* for 30-day survival of 0.77, 95% *CI* 0.62–0.97 (Table 2 and Fig. 2). Additionally, concomitant noradrenaline support and MCS usage were higher in milrinone patients. Post-PCI TIMI 3 flow was achieved more often, but they had lower LVEFs at the time of shock than the dobutamine group.

**Table 2:** Clinical course and outcomes

Characteristics	Unmatched cohort				Matched cohort		
	Milrinone, *N* = 247^a^	Dobutamine, *N* = 492^a^	*p***-**value	Missing %	Milrinone, *N* = 198^a^	Dobutamine, *N* = 198^a^	*p***-**value
Treatment characteristics during admission							
Noradrenaline	227 (91.9%)	391 (79.5%)	< 0.001	0	181 (91.4%)	161 (81.3%)	0.003
Adrenaline	44 (17.8%)	87 (17.7%)	0.965	0	32 (16.2%)	38 (19.2%)	0.429
Dopamine	3 (1.2%)	7 (1.4%)	1.000	0	3 (1.5%)	2 (1.0%)	1.000
Post-TIMI flow 3	188 (86.2%)	332 (79.0%)	0.027	14	145 (82.9%)	135 (79.9%)	0.478
Mechanical circulatory support	101 (41.1%)	160 (32.6%)	0.023	0.3	82 (41.6%)	66 (33.5%)	0.096
Laboratory values							
Troponins, ng/L^b^	5063.0 (1730.0, 10,000.0)	5716.0 (1630.0, 12,655.0)	0.362	15	5,717.0 (1,730.0, 10,000.0)	6,188.5 (1,732.0, 12,325.0)	0.570
CKMB, mcg/L^b^	305.5 (113.5, 594.0)	285.0 (83.0, 587.0)	0.451	50	335.0 (127.0, 608.0)	302.0 (100.0, 600.0)	0.527
Clinical outcomes							
LVEF	25.0 (25.0, 40.0)	33.0 (25.0, 40.0)	0.001	32	25.0 (25.0, 40.0)	30.0 (25.0, 40.0)	0.348
RVEF	40.0 (40.0, 55.0)	40.0 (40.0, 55.0)	0.391	57	40.0 (40.0, 55.0)	40.0 (40.0, 55.0)	0.672
Length of stay, days	7.0 (1.0, 17.0)	7.0 (1.0, 14.0)	0.662	20	8.0 (1.0, 18.0)	5.0 (1.0, 12.0)	0.159
Mortality at 30 days	125 (50.6%)	204 (41.5%)	0.018	0	92 (46.5%)	83 (41.9%)	0.362

*LVEF* left ventricular ejection fraction, *RVEF* right ventricular ejection fraction, *TIMI-flow* thrombolysis in myocardial infarction

^a^*n* (%); median (Q1; Q3)

^b^Highest level measured within 1–3 days after admission

### Outcomes after propensity score matching

**Fig. 3:**
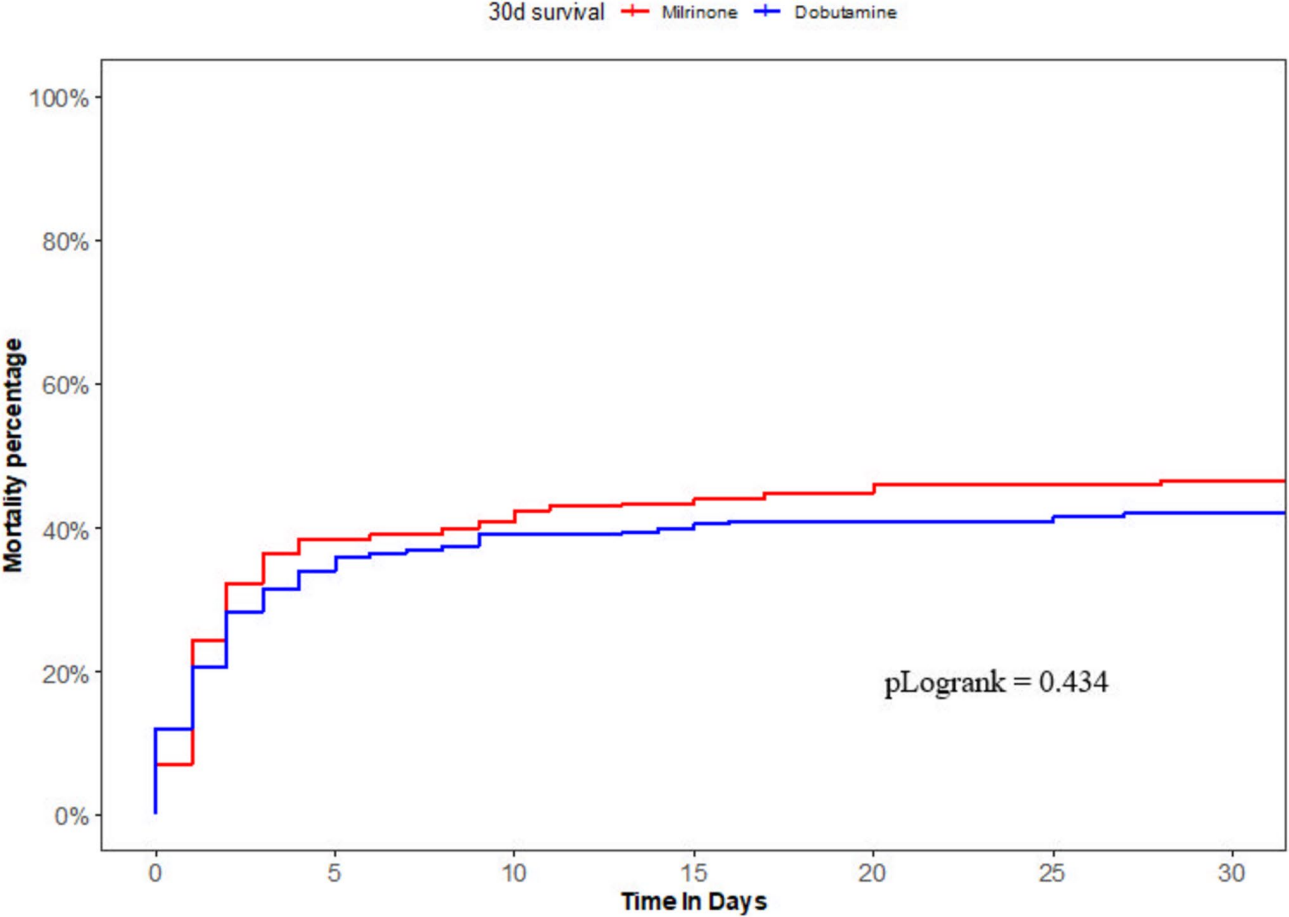
Kaplan–Meier curves of the matched cohort

After PSM, the 30-day mortality rates were similar between both groups (46.5% vs. 41.9%, *p* = 0.362), with an unadjusted *HR* for 30-day survival of 0.88, 95% *CI* 0.66–1.19 (Table 2 and Fig. 3). Consistently with the unmatched cohort, the milrinone group more frequently required additional noradrenaline. All other clinical course characteristics showed no significant differences between the milrinone and dobutamine group.

### Subgroup analyses

**Fig. 4:**
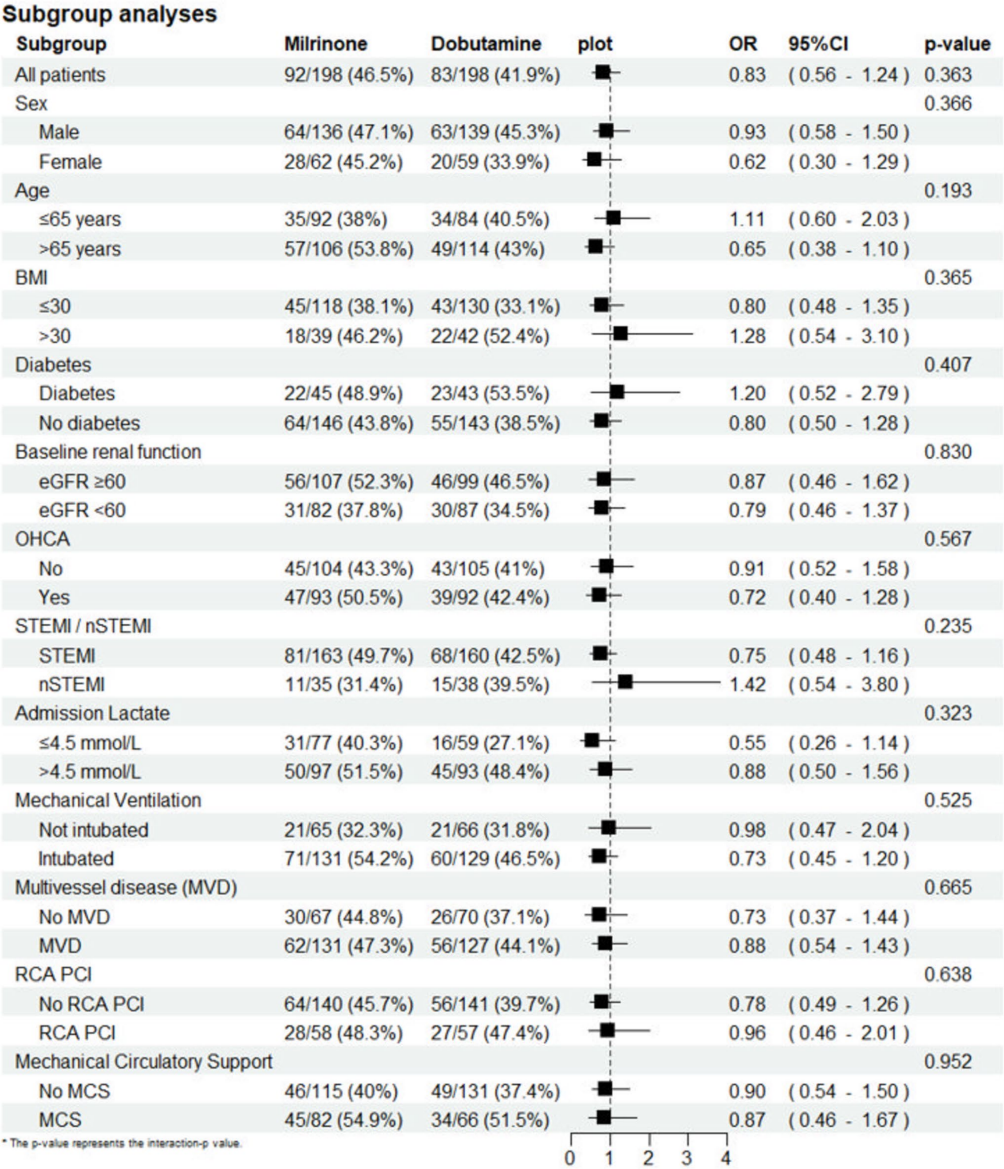
Subgroup analyses

Subgroup analyses, assessing the association between treatment with milrinone or dobutamine and 30-day mortality in defined subgroups, are displayed in Fig. 4. None of the individual subgroups exhibited a significant association with 30-day mortality, nor a significant interaction *p*-value.

### Additional analyses

The baseline table including patients treated with both milrinone and dobutamine, and with no inotrope, is provided in Supplemental Table S2. The Kaplan–Meier curves are provided in Supplemental Figure S1, showing higher 30-day mortality for patients treated with both milrinone and dobutamine and lower mortality for the ‘No inotrope’ group (*p*-Logrank < 0.001). Supplemental Figure S2 presents the Kaplan–Meier curves for patients without OHCA or IHCA of the matched cohort, showing no significant difference in 30-day mortality (unadjusted *HR* for 30-day survival 0.76, 95% *CI* 0.47–1.21).

Stratified by treatment group, Supplemental Tables 3 and 4 show the number of devices used and mortality by device type (Impella vs. ECMO). No significant differences in 30-day mortality were observed between the devices in either the unmatched or matched cohorts.

## Discussion

In this observational, real-world cohort — to our knowledge the largest available cohort comparing milrinone and dobutamine treatment in patients with AMI-CS undergoing PCI — we identified baseline characteristics associated with treatment with milrinone and dobutamine and investigated their effects on 30-day mortality using propensity-score matching methods.

In the unmatched cohort (*n* = 739), baseline, admission and catheterization laboratory characteristics differed significantly between the milrinone group (*n* = 247) and dobutamine group (*n* = 492). Upon admission, the milrinone patients presented with significantly higher heart rates, were more likely to present with NSTEMI and a consequently longer duration of symptoms before presentation, had higher levels of creatinine and more often experienced an IHCA. In the catheterization laboratory, they were more frequently intubated prior to CCU/ICU admission. Importantly, 30-day mortality (50.6% vs. 41.5%, *p* = 0.018), concomitant noradrenaline and MCS usage, and the ACCS-risk scores were higher in the milrinone group, indicating that these patients were more severely ill.

After PSM on 25 variables available at the catheterization laboratory, resulting in 198 patients per group, baseline, admission and catheterization laboratory characteristics became comparable. Also, 30-day mortality rates were now similar between the groups (46.5% vs. 41.9%, *p* = 0.362).

Both the European and American guidelines do not recommend a specific inotrope in AMI-CS treatment [1, 14]. The European guidelines recommend inotropes when needed to overcome the increased afterload often associated with noradrenaline use, and in patients with left ventricular systolic dysfunction, low cardiac output and low systolic blood pressure (e.g., < 90 mmHg) (14). The American guidelines suggest the addition of an inotropic agent in several CS subtypes [1]. This latter recommendation is based on a PSM analysis, comparing CS patients who received a vasopressor alone (noradrenaline, epinephrine, and dopamine) to those receiving a combination of a vasopressor and an inotrope, in which a lower short-term mortality was observed in the combination group [23]. The guidelines additionally recommend making the choice of inotrope based on the cause or presentation of CS; further considerations may include heart rate, systemic vascular resistance, renal function, prior β-blockade treatment and inotrope half-life [1].

Current evidence on the selection of inotrope is limited. The DOREMI trial is the only RCT (*n* = 192) comparing the effects of milrinone and dobutamine in CS patients, showing no significant differences in primary and secondary endpoints, including mortality [17]. However, only 65 patients (33.8%) of their cohort concerned AMI-CS patients [24]. Our study cohort and even the matched groups were substantially larger. Studies comparing milrinone to dobutamine have also been conducted in other patient populations, including a small RCT in patients awaiting cardiac transplant and an observational study of heart failure patients on chronic milrinone and dobutamine infusions [25, 26]. Both studies reported no difference in mortality. A meta-analysis on patients with CS or a low cardiac output state, including both the DOREMI trial in CS patients and the RCT on cardiac transplant patients, along with 9 observational studies, found no survival benefit for either inotrope in the combined RCT data [27]. However, a reduction in all-cause in-hospital mortality with milrinone was observed in the pooled data of the observational studies, most of which were of low methodologic quality. Moreover, a cohort study of patients with CS due to acute decompensated heart failure (ADHF-CS) showed a benefit of milrinone after PSM [28]. However, AMI-CS patients were excluded from this analysis and two-thirds of those included had long-standing heart failure before admission. This type of acute-on-chronic DHF-CS involves a subacute problem with a more gradual decline and chronic compensation mechanisms, contrasting the acute presentation of AMI-CS.

Inotrope selection in AMI-CS patients remains variable in clinical practice due to limited evidence. Theoretically, milrinone is often preferred in patients with pulmonary hypertension due to its potential lusitropic effects, which reduce pulmonary artery and central venous pressures more effectively than dobutamine [16, 29]. Milrinone may also be favored in patients at risk of tachyarrhythmias and myocardial ischemia, as it does not affect β-receptors and reduces myocardial oxygen consumption [15, 16, 30–32]. Milrinone also has a more pronounced vasodilatory effect, underscored by the higher use of noradrenaline in both the unmatched and matched cohorts, though no differentiation can be made whether noradrenaline had been started before or after milrinone or dobutamine initiation. Accordingly, in bradycardic and severely hypotensive patients, dobutamine may be the preferred inotrope. Also, in cases of acute kidney injury (AKI), where milrinone accumulation is a potential concern, dobutamine may be preferred agent [33]. A substudy of the DOREMI trial reported that milrinone was associated with lower risk of death in patients without AKI but not in those with AKI [34].

Of note, as a result of the PSM method, the milrinone patients were matched to the sicker dobutamine patients. Among dobutamine patients in the matched cohort, this is evident by the increase in heart rate, incidence of OHCA, and number of mechanically ventilated patients. As a result, 30-day mortality is higher in the dobutamine group within the matched cohort, whereas it remains unchanged in the milrinone group.

In the matched cohort, no association was found between treatment and mortality in any of the subgroups. Even among patients who underwent PCI of the right coronary artery (RCA), who might be expected to benefit most from milrinone, no difference was observed. However, RCA infarctions can also cause both severe hypotension and atrioventricular node block, in which case dobutamine may be preferred. These nuanced distinctions cannot be assessed with the existing data in this study. In the DOREMI trial, no mortality differences were observed in a small subgroup of patients with right ventricular involvement as well [17]. Importantly, in a sensitivity analysis excluding OHCA and IHCA patients, outcomes remained unchanged, suggesting that cardiac arrest did not drive the overall findings.

Lastly, although differences between Impella and ECMO devices in terms of hemodynamic profiles, complications and effectiveness might have influenced the comparison between milrinone and dobutamine, no significant differences in 30-day mortality were observed between devices in either the unmatched or matched cohorts. However, these findings are hypothesis generating due to low numbers of patients.

In this large national cohort of AMI-CS patients, no significant differences were found in 30-day mortality in a PSM cohort of milrinone and dobutamine patients. This is consistent with previously published small-sized data on patients with CS from various etiologies, heart failure patients, and patients awaiting cardiac transplant [17, 25–27]. These findings suggest that the choice of inotrope may continue to be tailored to individual patient characteristics at the discretion of the treating physician. Additionally, in our cohort lower unadjusted mortality was reported for patients who did not receive any inotrope. Accordingly, we await the results of the DOREMI-II trial, comparing milrinone and dobutamine to placebo, revising the foundational question whether inotropes are indicated at all in the CS population, as this has never been properly established [35].

### Limitations

Our study has several limitations inherent to observational cohort studies. One limitation is that we only had access to data on whether inotropes were administered during the period prior to PCI to 24 h post PCI. Therefore, this analysis lacks data on dosages, infusion rates, and duration. Another limitation is that, despite PSM, confounding by indication cannot be eliminated, as treatment decisions were made by treating physicians based on individual patient characteristics. Additionally, data on hemodynamic measurements and arrhythmic complications were missing. Furthermore, numbers in our subgroup and device comparison analyses were small and 95% *CI*s were not adjusted for multiple comparisons; therefore, these results should be considered hypothesis-generating rather than conclusive regarding treatment effects. Baseline characteristics and Kaplan–Meier curves of patients treated with both milrinone and dobutamine, and no inotrope, were exploratory; no conclusions should be drawn. Moreover, a center effect, with a specific preference for one inotrope over the other, could not be identified in this cohort. Lastly, data on LVEF, RVEF, and CKMB levels were incomplete.

## Conclusion

In this real-world propensity-matched cohort of patients with acute myocardial infarction-related cardiogenic shock, no significant difference in 30-day mortality was observed between patients treated with milrinone and dobutamine. Importantly, milrinone patients were sicker before matching, indicating that the choice of inotrope may be influenced by the severity of the illness. This comprehensive study suggests that the choice of inotrope may continue to be guided by individual patient characteristics.

## Supplementary Information

Below is the link to the electronic supplementary material.

ESM (DOCX 58.5 KB)

## Data Availability

The data that support the findings of this study are not publicly available because they are owned by third parties and restrictions apply to their availability. Data may be available from the third party upon reasonable request and with permission.

## Non Standard abbreviations and acronyms

AMI-CS: acute myocardial infarction-related cardiogenic shock
MCS: mechanical circulatory support
TIMI: thrombolysis in myocardial infarction
MVD: multivessel disease
LVEF: left ventricular ejection fraction
RVEF: right ventricular ejection fraction
