# Supplementary material for: Milrinone versus dobutamine in acute myocardial infarction-related cardiogenic shock; a propensity score matched analysis
Source: Clin Res Cardiol. 2025 Sep 3;114(10):1414–26. doi: 10.1007/s00392-025-02742-0 (PMC12460367; doi:10.1007/s00392-025-02742-0)
Supplement: Supplementary file 1 — ESM (DOCX 58.5 KB) [file 392_2025_2742_MOESM1_ESM.docx]

**Supplemental material**

Supplemental Table 1: Covariates selected for the propensity score matching model

Supplemental Table 2: Baseline table unmatched cohort including patients who received both milrinone and dobutamine, and no inotrope

Supplemental Figure 1: Kaplan Meier curves unmatched cohort including patients who received both milrinone and dobutamine, and no inotrope

Supplemental Figure 2: Kaplan Meier curves matched cohort of patients without OHCA or IHCA

Supplemental Table 3: Types of mechanical circulatory support in matched and unmatched milrinone and dobutamine groups

1. All separate groups, B) Combinations used

Supplemental Table 4: 30-day mortality by device (Impella vs. ECMO) within milrinone and dobutamine groups

Supplemental Table 1. Covariates selected for the propensity score matching model

| **Baseline characteristics** |
| --- |
| Age |
| Sex |
| BMI |
| Diabetes |
| Prior coronary event (PCI or CABG) |
| **Admission characteristics** |
| MAP |
| Heart rate |
| STEMI or non-STEMI |
| Short duration of symptoms (<3 hours) |
| Presentation with an OHCA |
| Witnessed OHCA |
| OHCA duration (<30 minutes or ≥30 minutes) |
| IHCA prior to PCI |
| Cardiac arrest at the catheterization laboratory |
| Intubation before coronary care unit (CCU)/ intensive care unit (ICU) admission |
| **Laboratory values at admission** |
| Lactate levels |
| Hemoglobin levels |
| Glucose levels  Creatinine |
| **Angiography** |
| Target vessel treated with PCI |
| Preprocedural TIMI-flow (0-1 or 2-3) |
| Multivessel disease |

*BMI = body mass index; CABG = coronary artery bypass grafting; CCU = coronary care unit; ICU = intensive care unit; IHCA = in-hospital cardiac arrest; MAP = mean arterial pressure; OHCA = out-of-hospital cardiac arrest; PCI = percutaneous coronary intervention; TIMI = Thrombolysis in Myocardial Infarction*

Supplemental Table 2. Baseline table unmatched cohort including patients who received both milrinone and dobutamine, and no inotrope

| **Characteristics** | | **Milrinone**  N = 247^a^ | **Dobutamine**  N = 492^a^ | **Milrinone and Dobutamine**  N = 177^a^ | **No inotrope**  N = 848^1^ | **Missing %** |
| --- | --- | --- | --- | --- | --- | --- |
| **Baseline characteristics** | |  |  |  |  |  |
| Age | | 66.0 (12.0) | 67.1 (12.1) | 65.5 (12.1) | 66.8 (12.6) | 0 |
| Gender, Male | | 177 (71.7%) | 339 (68.9%) | 128 (72.3%) | 619 (73.0%) | 0 |
| BMI | | 27.7 (5.0) | 26.6 (4.5) | 26.8 (4.1) | 26.4 (4.6) | 14 |
| Diabetes | | 59 (24.6%) | 108 (23.0%) | 35 (20.8%) | 147 (17.9%) | 4.1 |
| Prior event | | 85 (37.1%) | 126 (28.8%) | 64 (37.0%) | 200 (25.1%) | 8.3 |
| **Admission characteristics** | |  |  |  |  |  |
| MAP (mmHg) | | 77.7 (22.6) | 77.8 (24.1) | 75.9 (22.1) | 77.7 (24.6) | 15 |
| Heart rate (bpm) | | 95.9 (27.5) | 82.5 (27.6) | 91.9 (28.0) | 77.1 (28.7) | 16 |
| STEMI (vs. NSTEMI) | | 178 (72.1%) | 425 (86.4%) | 138 (78.0%) | 764 (90.1%) | 0 |
| Symptoms > 3 hours | | 120 (56.1%) | 169 (40.0%) | 80 (52.6%) | 285 (38.7%) | 14 |
| OHCA | | 116 (47.2%) | 201 (41.0%) | 57 (32.2%) | 228 (26.9%) | 0.3 |
| Witnessed arrest | | 80 (33.1%) | 167 (34.6%) | 47 (27.3%) | 182 (21.7%) | 2.2 |
| Resuscitation duration ≥ 30 minutes | | 32 (14.0%) | 55 (11.7%) | 21 (12.6%) | 13 (1.6%) | 5.2 |
| IHCA | | 16 (6.5%) | 28 (5.7%) | 15 (8.5%) | 35 (4.1%) | 0.3 |
| Intubated before CCU/ICU admission | | 168 (68.6%) | 274 (56.4%) | 119 (67.6%) | 241 (28.7%) | 1.0 |
| **Laboratory values at admission** | | | | |  |  |
| Lactate (mmol/l) | | 5.6 (2.6, 9.5) | 6.0 (3.0, 9.6) | 5.7 (2.7, 9.5) | 3.8 (1.9, 6.6) | 21 |
| Hemoglobine (mmol/l) | | 8.0 (1.6) | 8.3 (1.4) | 8.2 (1.4) | 8.3 (1.3) | 4.0 |
| Glucose (mmol/l) | | 12.8 (8.6, 18.7) | 13.4 (9.4, 17.8) | 12.9 (9.9, 18.0) | 10.1 (8.0, 13.2) | 9.6 |
| Creatinine (μmol/l) | | 111.0 (88.0, 139.0) | 103.0 (82.0, 123.0) | 108.0 (88.0, 142.0) | 93.0 (77.0, 115.0) | 9.4 |
| **Angiography** | |  |  |  |  |  |
| Left main | | 47 (19.0%) | 76 (15.4%) | 34 (19.2%) | 68 (8.0%) | 0 |
| LAD | | 110 (44.5%) | 214 (43.5%) | 84 (47.5%) | 300 (35.4%) | 0 |
| RCX | | 71 (28.7%) | 92 (18.7%) | 60 (33.9%) | 152 (17.9%) | 0 |
| RCA | | 74 (30.0%) | 142 (28.9%) | 48 (27.1%) | 351 (41.4%) | 0 |
| Multivessel disease | | 164 (66.4%) | 299 (61.1%) | 123 (69.9%) | 161 (22.4%) | 0.4 |
| Pre-TIMI flow 2-3 | | 66 (31.1%) | 96 (23.7%) | 36 (23.7%) | 497 (59.0%) | 16 |
|  | ^a^Mean (SD); n (%); Median (Q1, Q3)  *BMI = body mass index; CCU = critical care unit; ICU = intensive care unit; IHCA = in-hospital cardiac arrest; LAD = left anterior descending artery; MAP = mean arterial pressure; NSTEMI = non-ST-elevation myocardial infarction; OHCA = out-of-hospital cardiac arrest; RCA = right coronary artery; RCX = ramus circumflex; STEMI = ST-elevation myocardial infarction; TIMI-flow = Thrombolysis in Myocardial Infarction* | | | | | |

Supplemental Figure 1. Kaplan Meier curves unmatched cohort including patients who received both milrinone and dobutamine, and no inotrope

pLogrank <0.001


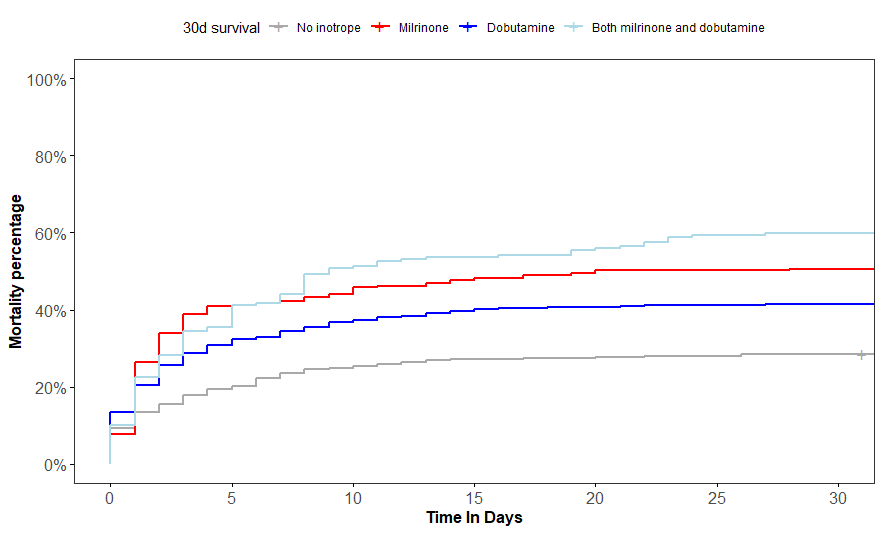


pLogrank <0.001

Supplemental Figure 2. Kaplan Meier curves matched cohort of patients without OHCA or IHCA


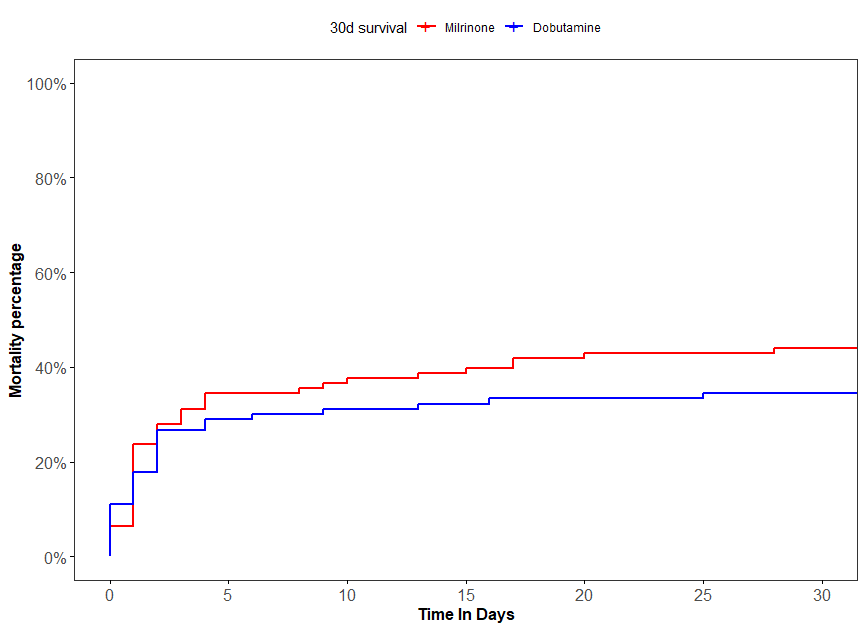


pLogrank = 0.239

Supplemental Table 3: Types of mechanical circulatory support in matched and unmatched milrinone and dobutamine groups

1. Separate MCS groups, B) Combinations used

A)

|  | Unmatched | | Matched cohort | |
| --- | --- | --- | --- | --- |
|  | Milrinone (n=247) | Dobutamine (n=492) | Milrinone (n=198) | Dobutamine (n=198) |
| IABP | 47 (19.1%) | 82 (16.7%) | 37 (18.8%) | 32 (16.2%) |
| Impella | 23 (9.3%) | 27 (5.5%) | 19 (9.6%) | 9 (4.6%) |
| ECMO | 14 (5.7%) | 20 (4.1%) | 12 (6.1%) | 9 (4.6%) |
| Combinations or else | 17 (16.8%) | 31 (19.4%) | 14 (17.1%) | 16 (24.2%) |

B)

|  | Unmatched | | Matched cohort | |
| --- | --- | --- | --- | --- |
|  | Milrinone (n=247) | Dobutamine (n=492) | Milrinone (n=198) | Dobutamine (n=198) |
| ECMO + IABP | 10 (5.1%) | 13 (6.6%) | 12 (4.9%) | 24 (4.9%) |
| ECMO + Impella | 4 (2.0%) | 2 (1.0%) | 4 (1.6%) | 5 (1.0%) |
| Impella + IABP | 0 (0.0%) | 0 (0.0%) | 1 (0.4%) | 1 (0.2%) |
| Else | - | 1 (0.5%) | - | 1 (0.2%) |

Supplemental Table 4. 30-day mortality by device (Impella vs. ECMO) within milrinone and dobutamine groups

Unmatched cohort, Milrinone patients, p-value 0.487;

| Group | N Patients | N Deaths | Mortality - % |
| --- | --- | --- | --- |
| ECMO | 14 | 8 | 57.1 |
| Impella | 23 | 17 | 73.9 |

Unmatched cohort, Dobutamine patients, p-value 0.935;

| Group | N Patients | N Deaths | Mortality - % |
| --- | --- | --- | --- |
| ECMO | 20 | 10 | 50 |
| Impella | 27 | 12 | 44.4 |

Matched cohort, Milrinone patients, p-value 0.705;

| Group | N Patients | N Deaths | Mortality - % |
| --- | --- | --- | --- |
| ECMO | 12 | 7 | 58.3 |
| Impella | 19 | 13 | 68.4 |

Matched cohort, Dobutamine patients, p-value 0.637;

| Group | N Patients | N Deaths | Mortality - % |
| --- | --- | --- | --- |
| ECMO | 9 | 3 | 33.3 |
| Impella | 9 | 5 | 55.6 |
